# Estimating the relative importance of demic and cultural diffusion in the spread of the Neolithic in Scandinavia

**DOI:** 10.1098/rsif.2018.0597

**Published:** 2018-11-21

**Authors:** Joaquim Fort, Maria Mercè Pareta, Lasse Sørensen

**Affiliations:** 1 Complex Systems Laboratory, University of Girona, C/. Maria Aurèlia Capmany 61, 17003 Girona, Spain; 2 Catalan Institution for Research and Advanced Studies (ICREA), C/. Lluís Companys 23, 08010 Barcelona, Spain; 3 Ancient Cultures of Denmark and the Mediterranean, The National Museum of Denmark, Frederiksholms Kanal 12, 1220 Copenhagen K, Denmark

**Keywords:** neolithic transition, spread rate, demic diffusion, cultural diffusion, Scandinavia

## Abstract

Using a database of early farming sites in Scandinavia, we estimate that the spread rate of the Neolithic was in the range 0.44–0.66 km yr^−1^. This is substantially slower (by about 50%) than the rate in continental Europe. We interpret this result in the framework of a new mathematical model that includes horizontal cultural transmission (acculturation), vertical cultural transmission (interbreeding) and demic diffusion (reproduction and dispersal of farmers). To parametrize the model, we estimate reproduction rates of early farmers using archaeological data (sum-calibrated probabilities for the dates of early Neolithic Scandinavian sites) and use them in a wave-of-advance model for the first time. Comparing the model with the archaeological data, we find that the percentage of the spread rate due to cultural diffusion is below 50% (except for very extreme parameter values, and even for them it is below 54%). This strongly suggests that the spread of the Neolithic in Scandinavia was driven mainly by demic diffusion. This conclusion, obtained from archaeological data, agrees qualitatively with the implications of ancient genetic data, but the latter are yet too few in Scandinavia to produce any quantitative percentage for the spread rate due to cultural diffusion. We also find that, on average, fewer than eight hunter–gatherers were incorporated in the Neolithic communities by each group of 10 pioneering farmers, via horizontal and/or vertical cultural transmission.

## 1. Introduction

In Europe, agriculture and stockbreeding (i.e. the Neolithic) arrived from the Near East and replaced previous economic and social systems based on hunting and gathering (Mesolithic). Two possible mechanisms (or a combination of them) have been proposed as being responsible for the spread of the Neolithic. The first one, demic diffusion, refers to the dispersal of farming populations. The second one, cultural diffusion, refers to the incorporation of hunter–gatherers into the farming communities (via interbreeding and/or acculturation). Views on the relative importance of demic and cultural diffusion in the spread of the Neolithic in Europe have evolved in parallel to the availability of new data and methods, as we summarize below.

Almost 50 years ago, Ammerman and Cavalli-Sforza estimated that the Neolithic spread across Europe at a speed of about 1 km yr^−1^ [1]. They noted that this value agrees with that predicted by a purely demic model and proposed that the spread of the Neolithic across most of Europe had been mainly demic. However, they suggested that there had also been some degree of cultural diffusion, and that it could have generated genetic clines [1,2]. Some years later, the similarity between a map of the first principal component of classical genetic markers (blood groups and other proteins) in the present Europeans and an interpolation map of radiocarbon dates [3] was interpreted as supporting this hypothesis of mainly demic diffusion [4]. By then, it was possible to study genes only at the level of proteins.

It is of interest to understand intuitively why the spread rate was only about 1 km yr^−1^. The reason for this is that the speed of demic diffusion is inherently limited by demographic parameter values. Indeed, no matter how fast a population reproduces, it cannot spread demically at a speed faster than the longest dispersal distance of individuals per generation (Δ_max_) divided by the corresponding time interval *T* (the generation time) [5]. This is rather obvious intuitively. As we will discuss in detail in §4, ethnographic data suggest that this maximum speed is 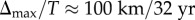 or about 3 km yr^−1^ (we mention that this value of Δ_max_ is valid for inland travel). This shows that a speed of about 1 km yr^−1^ agrees, concerning the order of magnitude, with the maximum possible speed for inland human demic expansions.

In the last decade of the last century, it became possible to study directly what genes are made of, namely DNA, and this led to a new consensus in the genetics community. Indeed, during the first years of the present century, it became widely accepted that cultural diffusion had been more important than demic diffusion [6–8]. But, again, all of that work was based on analysing the genetics of *modern* populations.

In the first decade of this century, *ancient* DNA studies changed this view again. Such studies were at first based only on haplogroups, and they already indicated a substantial genetic turnover at the arrival of the Neolithic [9]. In parallel, the combination of archaeological data with mathematical modelling led to the proposal (without using any genetic data) that demic diffusion had a more important effect than cultural diffusion on the spread rate of the Neolithic in Europe [5].

Recently, genome-wide ancient data have led to more detailed results than approaches at the haplogroup level and led to the conclusion that demic diffusion was much more important than cultural diffusion. For example, Mathieson *et al.* [10] estimated that early Neolithic farmers from Germany, Hungary and Spain had a genomic Anatolian component larger than 90%, and the rest (below 10%) was identified as hunter–gatherer ancestry. This implies that the *modern* DNA work summarized above [6,11,12] had erroneously identified the non-Neolithic component in the modern European gene pool as a Palaeolithic one. By contrast, *ancient* DNA [10,13,14] indicates that the non-Neolithic component is mainly due to post-Neolithic migrations [8,15–17]. Owing to these results, nowadays the genetic consensus is that demic diffusion was more important than cultural diffusion in the spread of the Neolithic in Europe.

Genome-wide results also indicate that early Neolithic farmers from Iberia (Epicardial culture), central Europe (LBK culture), the Balkans and Anatolia [18], as well as those from Britain [19], are all closely related. This provides strong support for a single migration from Anatolia. Similarly, genome-wide ancient DNA has shown that early Neolithic farmers in Scandinavia have mainly the same ancestry as those in Central Europe and the Near East [14,20,21], but early Scandinavian farmers display more admixture with hunter–gatherers than early Central European farmers [14,22]. Then the question arises of to what extent cultural diffusion could have partially driven the Neolithic spread in Scandinavia.

In spite of the unquestionable importance of genome-wide studies, it should be stressed that they do not yield any quantitative estimation of the relative importance of demic and cultural diffusion *on the spread rate* of the Neolithic (note that the spread rate, or front speed, is the distance advanced by the Neolithic front per unit time, and is measured in kilometres per year). Indeed, genome-wide studies estimate fractions 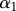, 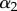, … , 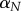 (due to *N* presumed populations) of the genetic drift *f*_4_ [10] (defined as a variance of allele frequencies [23]). But, there is no theory relating the fractions of genetic drift to the percentages of demic and cultural diffusion *on the spread rate* of the Neolithic wave of advance (and the same happens with other genetic methods, e.g. the fractions of the genome estimated by admixture analysis [24]). In other words, knowing, for example, the fraction 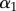 of the *genetic* Anatolian component of Scandinavian early farmers does not make it possible to know the effects (percentages) of demic and cultural diffusion *on the Neolithic spread rate* in Scandinavia. Therefore, as stressed previously [5], the relative importance of demic and cultural diffusion on the *genetic pool* and on the *spread rate* need not be the same. These two problems are related to each other, but only qualitatively, in the sense that if fewer hunter–gatherers were incorporated into the farming communities, then obviously the genetic Anatolian component 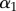 of Scandinavian early farmers would be higher and the cultural effect on the spread rate would be lower. But, they are not quantitatively related. For example, there is no proof that if the genetic Anatolian component 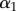 is above 50% then the cultural effect *on the spread rate* will be below 50%. Hence, a mainly demic process concerning the genetics is not necessarily a mainly demic process concerning archaeology (spread rate). These are two different problems, and they require different methods of analysis [5]. In this paper, we deal with the second problem by using archaeological data to estimate the spread rate of the Neolithic in Scandinavia. We also compare it with a new wave-of-advance model to estimate to what extent demic and/or cultural diffusion could have been responsible for it.

Several authors have investigated how, when and why agrarian societies spread during the late 5th and early 4th millennium BC across Scandinavia [25,26]. Proposed reasons for the adoption of agrarian practices in South Scandinavia concentrate on population growth, resource availability caused by climate changes, social changes within societies, or a combination of all three [25]. Most researchers tend to prefer one explanation over another, but currently no dominant reason is preferred. The perception of who were the primary carriers of agrarian knowledge and practices also varies. Until recently, the three main hypotheses were migration, indigenism and integration. The migration hypothesis (purely demic diffusion) argues that agriculture was introduced by a swift process of a smaller or larger migration lasting only a few generations at each location. Here, it is the migrating farmers who are the primary carriers of agrarian technologies. On the other hand, the hypothesis of indigenism (purely cultural diffusion) argues that the introduction of agrarian technologies is a gradual process, lasting several hundred years, in which the hunter–gatherers are the primary carriers of agrarian technologies (which thus spread as an idea between humans). As mentioned above, this second proposal (purely cultural diffusion) has now been ruled out by ancient genomic data [14,20,21]. Finally, supporters of the integration hypothesis (demic–cultural diffusion) defend a combination of the first two hypotheses, but here there is still no agreement about how big a role the local hunter–gatherers played in this spread of agrarian technologies. A new methodological approach, previously not used on any Scandinavian material, is applied in the present paper to estimate the relative importance of demic and cultural diffusion on the spread rate of the Neolithic in Scandinavia.

The key issues that we address in the present paper are the following. Firstly, we estimate quantitatively the spread rate of the Neolithic in Scandinavia and find that it was substantially slower than in most of Europe. This is quite unexpected, given the widespread notion that the Neolithic spread in Scandinavia was extremely rapid [27,28]. Secondly, using a new mathematical wave-of-advance model, we try to understand the reason why the Neolithic spread rate in Scandinavia was so slow. Thirdly, we attempt to determine whether in Scandinavia (similarly to most of Europe [5]) demic diffusion had a more important effect than cultural diffusion *on the Neolithic spread rate* or not.

Scandinavia is a huge region (with distances up to about 2000 km), and this makes it possible to perform a statistically significant estimation of the Neolithic spread rate (as shown in §2).

Similar to Fort [5], our approach is based on using archaeological data to estimate the spread rate and comparing it with a mathematical wave-of-advance model. The two main differences between the present paper and Fort [5] are that here we consider Scandinavia (whereas in Fort [5] we considered all of Europe except Scandinavia), and that here we derive and apply a new mathematical model including both acculturation and interbreeding (whereas in Fort [5] we included only acculturation, and in Fort [29] we included only interbreeding). Acculturation and interbreeding are of interest in anthropology, archaeology, history and genetics. However, we think that, with appropriate modifications, our equations could also be useful in other disciplines where front propagation with interaction is important, including linguistics (language competition), ecology (predator–prey interactions and ecological competition), medicine (the spread of diseases and epidemics) and the physical sciences (impurities and/or porosity effects on chemical and combustion front propagation).

Another novelty of the present paper is that we apply, for the first time, estimations of growth (or reproductive) rates obtained from archaeological (rather than ethnographic [5]) data to a wave-of-advance model, which in our opinion is a relevant methodological advance over previous work [5].

## 2. Estimating the spread rate

Recently, it has been shown that a rapid warming took place in Scandinavia around 6000 cal yr BP, which improved environmental conditions and extended the growing season of domestic crops, leading at about the same time to a farmer population boom (Funnel Beaker culture) and the spread of the Neolithic northwards [30]. In this section, we estimate quantitatively the rate of this spread (in kilometres per year).

**Figure 1.**
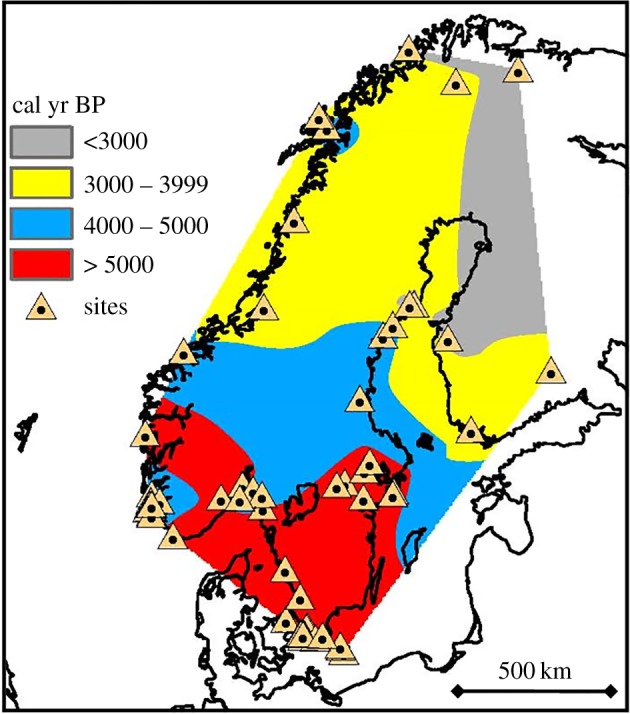
Map of Scandinavia with the sites in our database shown as triangles (electronic supplementary material, Info. database). Colours have been obtained by interpolation, and correspond to the areas covered by the Neolithic wave of advance every 1000 years.

We compiled a database of early Neolithic sites with cereals in Scandinavia. We consider only the oldest date for each site, which is the best available estimation of the local arrival date of the Neolithic (with the data known at present). We include the database as electronic supplementary material. It also contains the sources where the C-14 dates were originally reported (see also [25,26,31,32] and references therein). Calibration has been carried out using OxCal v4.3.2. The sites are shown as triangles in figure 1, where we also include the isochrones obtained by natural neighbour interpolation (other interpolation methods, e.g. kriging, yield similar results). The isochrones in figure 1 indicate a clear northward spread. Unfortunately, the database has only 70 sites, so we can only estimate an average rate (but not different rates in different regions). The oldest site in our database is Oxie (dated 6150 cal yr BP). It is one of the several superimposed triangles on the southwest of the southern tip of Sweden, as shown in figure 1. We have considered this site as a plausible origin for the diffusion of the Neolithic northwards because it is the oldest site in the database. Of course, this does not mean that we consider that the Neolithic could not enter Sweden via other places. It only means that Oxie is probably located in the region where the Neolithic entered Sweden, and in this sense it is reasonable to use Oxie (or any other nearby location) to compute the distances traversed by the Neolithic wave of advance. In fact, using other reasonable origins yields the same conclusions (see below and electronic supplementary material, especially §S3). Thus, we have computed the great-circle distance (defined as the smallest distance on the Earth's surface, considered as a sphere) from Oxie to each site in the database (the distances are also included in the electronic supplementary material). The equation used to compute great-circle distances is included in the electronic supplementary material, §S4, which also contains the statistical method used to estimate the spread rate and its error.

**Figure 2.**
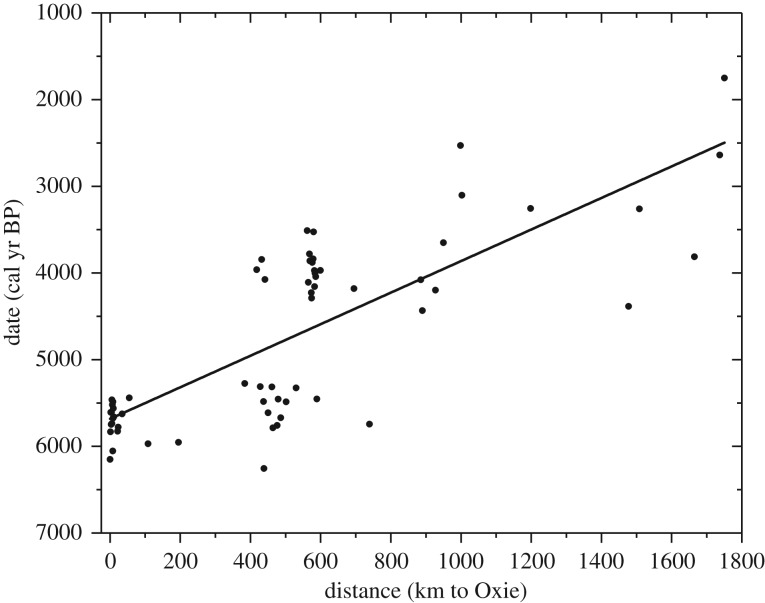
Linear regression fit of calibrated dates of early Neolithic sites in Scandinavia versus great-circle distances relative to the site of Oxie in southern Sweden (which is the oldest site in the database). According to this linear fit (solid line), the Neolithic spread in Scandinavia with a rate in the range 0.44–0.66 km yr^−1^ with 95% CL. The correlation coefficient is rather high (*r* = 0.77), which implies that a linear fit is reasonable. *N* = 63 sites.

A problem arises due to the fact that sea travels are not strictly included in the mathematical wave-of-advance model (§3) that we shall use to interpret the spread rate. The reason for this is that only models assuming that humans can live everywhere on the landscape make it possible to derive an equation for the spread rate (otherwise the double integral in the electronic supplementary material, equation (S14), for the spread rate cannot be solved). Therefore, in principle the model is not strictly appropriate to describe situations with sea travel (unless the sea distances involved are small). For this reason, we think that, to obtain a precise estimation of the spread rate, it is reasonable to exclude from the calculations the four sites in Denmark and the three sites in Finland (although, in fact, including these leads to rather similar results; see below). Figure 2 includes 63 sites, i.e. the 70 sites in our database except the four sites in Denmark and the three sites in Finland (the last three are located in the middle right of figure 2). As usual [33], we performed a time-versus-distance linear regression (figure 2) because distances are, in principle, known more precisely than dates since the latter include, among others, errors due to calibration and because not all sites have been discovered and dated (so we cannot be sure of the arrival date of the Neolithic at each location). According to the linear regression in figure 2, the Neolithic spread across Scandinavia with a rate in the range 0.44–0.66 km yr^−1^, with a 95% confidence level (CL). The correlation coefficient is rather high (*r* = 0.77), which implies that a linear fit with origin at the site of Oxie is reasonable. A significant trend is also implied by the fact that the slope is very highly significantly different from zero (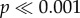). In this paper, we shall refer to this result, i.e. 0.44–0.66 km yr^−1^, as the ‘observed’ spread rate (in the sense that it has been obtained from archaeological data). We note that this rate is substantially slower (about half) than the rate for the spread of the Neolithic across Europe, namely 0.9–1.0 km yr^−1^, which was estimated previously by the same method (namely, a linear fit to calibrated dates versus great-circle distances) [34]. In fact, an average rate of Neolithic spread over Europe of about 1 km yr^−1^ was well established many years ago [1]. In the next sections, we will compare the observed spread rate in Scandinavia (0.44–0.66 km yr^−1^) with that predicted by a mathematical wave-of-advance model (§3) using realistic parameter values, to understand why the spread of the Neolithic across Scandinavia was so slow. We shall also use the observed range and the model to make a quantitative estimation of the effects of demic and cultural diffusion on the Neolithic spread rate in Scandinavia.

We mention that other analyses are possible, but lead to similar results. For example, excluding only the four sites in Denmark (*N* = 66 sites) leads to a spread rate of 0.42–0.64 km yr^−1^ (95% CL), *r* = 0.78 and 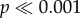. Alternatively, if all 70 sites are included, the spread rate is 0.42–0.62 km yr^−1^ (95% CL), *r* = 0.79 and 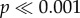. Also, instead of Oxie, a different old site could be used as a distance origin. For example, there are four sites in Denmark in our database. Three of them are located on the small island of Bornholm, about 100 km offshore from the south of the Swedish mainland (these are the three southernmost triangles in figure 1) and the other one is Ullerødgård (5614 cal yr BP). If we use Ullerødgård as a distance origin, we obtain 0.41–0.61 km yr^−1^ (95% CL), *r* = 0.78 and 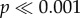 (*N* = 70), which are again similar results to those from our three analyses above. As explained above, owing to the features of the analytical model (§3), we think that it is more reasonable to use the range obtained by neglecting sites affected by sea travel, i.e. using *N* = 63 sites (0.44–0.66 km yr^−1^). However, we stress that the conclusions would not change by using the spread rate obtained from any of the other three analyses summarized above, or similar ones. Moreover, in the electronic supplementary material, we show that the conclusions of this paper would be the same if we took into account that distances are affected by landscape, vegetation, etc. (§S1), we justify the linear model compared with nonlinear ones (§S2), and we check that Oxie (or nearby origins) fit the data best (§S3).

## 3. Mathematical model

Until recently, mathematical models used to interpret Neolithic spread rates included only demic diffusion, i.e. the dispersal and reproduction of farmers. The first mathematical model, due to Fisher, was applied to the Neolithic by Ammerman and Cavalli-Sforza [2,4]. Later, more accurate demic models were derived by generalizing that model to two dimensions [35] and by taking into account the cohabitation time between newborn children and their parents [35,36] as well as the dependence of the dispersal probability on distance (dispersal kernel) [36,37]. All of these models are purely demic, i.e. they do not include cultural transmission.

In recent years, cultural transmission theory [38] has been incorporated to build demic–cultural models of Neolithic spread. As mentioned in §1, in our context, cultural transmission refers to the incorporation of hunter–gatherers into the farming communities. There are two main types of cultural transmission [38]. The first one, horizontal transmission, is the acculturation of hunter–gatherers (they acquire the domesticates and knowledge from neighbouring farmers and become farmers themselves). The second type, vertical transmission, is due to interbreeding between hunter–gatherers and farmers (their children are farmers according to ethnographic observations [4,39]). Previous work on demic–cultural wave-of-advance propagation models has included only either horizontal [5] or vertical [29] transmission. By contrast, here we present a new model that includes both horizontal and vertical transmission. The model is based on the following two equations for the population densities of farmers *N* and hunter–gatherers *P* at position (*x,y*) and time *t*:3.1
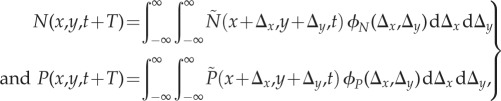
where *T* is the generation time, defined as the mean age difference between a parent and her/his children (and assumed to be approximately the same for both populations). 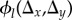 is the dispersal kernel of population *l* = *N*, *P*, defined as the probability to move distances 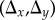 per generation. Equations (3.1) simply state that some of the individuals living at position (*x,y*) at time *t* + *T* may, in principle, have arrived from any other position 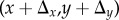. In the case of farmers (*N*), they can have appeared at 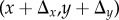 due to reproduction [*R_T_*] of farmers living at 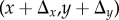 a generation before (time *t*), or they can be former hunter–gatherers who have become farmers via horizontal [*H_T_*] and/or vertical [*V_T_*] transmission. These increases, and the corresponding decreases for hunter–gatherers, are taken into account by 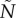 and 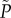 in equations (3.1), which are defined as3.2
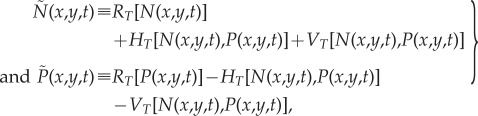
where the first terms on the right-hand side are the new population densities due to logistic net reproduction (with initial growth rates *a_l_* and carrying capacities *K_l_*) during the time interval *T*, namely (see §S5 in the electronic supplementary material)3.3
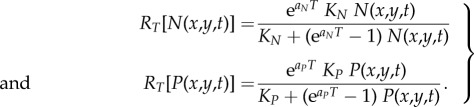
The second terms on the right-hand side of equations (3.2) correspond to horizontal cultural transmission and can be written as [5] (see §S5 in the electronic supplementary material for a derivation)3.4
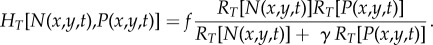


Note that horizontal transmission is driven by parameters *f* and *γ* [5].

Finally, the last terms in equation (3.2) correspond to vertical cultural transmission and are given by [29,38] (see §S5 in the electronic supplementary material for a derivation)3.5
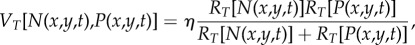
so that vertical transmission is driven by parameter *η*.

Equations (3.1)–(3.5) assume that reproduction takes place, followed by cultural transmission and then dispersal. However, the spread rate would be the same (namely equation (3.6)), whatever the order of these events.

The spread rate of the farming waves of advance driven by equations (3.1)–(3.5) is (electronic supplementary material, §S5)3.6
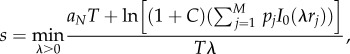
where3.7
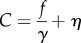
can be considered as a measure of the joint intensity of horizontal and vertical cultural transmission, because *C* is equal to the mean number of hunter–gatherers converted into farming (by horizontal and/or vertical transmission) per pioneering farmer and generation (electronic supplementary material, §S5). *p_j_* is the probability for farmers to disperse a distance *r_j_* (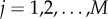) and 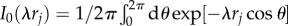 is the modified Bessel function of the first kind and order zero.

## 4. Parameter values

Previous applications of wave-of-advance models to the spread of the Neolithic have been based on ethnographic estimations for the growth rate *a_N_* of pre-industrial farmers who settled in empty space [4,5,37]. However, Scandinavia has high latitudes and, at least in present populations, it has been observed that increasing latitude is correlated with decreasing fertility [40]. In the absence of ethnographic estimates of the growth rate of pre-industrial farmers who settled in empty space in Scandinavia, one approach could be to apply such a correction to ethnographic estimates for lower latitudes, but this would obviously introduce an additional source of error to that due to the use of ethnographic rather than archaeological data. Fortunately, at present it is possible to follow a much more direct approach, i.e. one based directly on archaeological data. Indeed, it has often been proposed that summed probability distributions of radiocarbon dates can be used as estimations of relative population sizes, and authors who have computed such probability distributions have detected a rise in several Scandinavian regions at about the time when the Neolithic arrived (around 6000 cal yr BP), including in their databases both Neolithic and Mesolithic sites [30,41–43]. However, in the present paper, we need to estimate the initial growth rate *a_N_* of farmers, so obviously we have to consider only Neolithic sites. Such data were reported by Hinz *et al.* [44] for the Funnel Beaker culture in several regions of Scandinavia. They published two kinds of plots for the probability versus time. The first plot for each region (fig. 3 in [44]) is based on the whole set of dates in their database (from settlements, enclosures, graves and ritual depositions). The second plot, also for each region (fig. 4 in [44]), is based on data from settlements only. Each plot is different, but in all cases there are substantial increases in the probability near the arrival of the Neolithic (at around 6000 cal yr BP). The duration of each increase is usually about 100–200 yr. Accordingly, we consider a time interval of 100 yr (or about three generations [45]) to perform an estimation of the growth rate *a_N_* from each plot.

The ideal approach would be to fit an exponential function to each dataset, but the large time span covered in the plots (2000 yr) in [44] is much larger than 100 yr, so unfortunately we do not have detailed enough data to attempt a fit for a time interval of 100 yr. However, we can easily use each of those plots to perform estimations of the relative population numbers at the start of the increase (*N*_0_) and 100 yr later (*N*). From this, we can estimate the value of *N*/*N*_0_ for each plot by assuming an exponential growth, 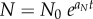 (which is a good approximation to logistic growth for low population numbers, i.e. during the first generations after the arrival of farmers; see electronic supplementary material, §S5, or [46]). Thus, the initial growth rate of farmers *a_N_* can be estimated as4.1
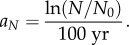
We have applied equation (4.1) to obtain estimations of *a_N_*, one from each plot. The results are shown in table 1, and most of them are of the order of 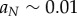 yr^−1^. The complete range for *a_N_* implied by the 14 values in table 1 is 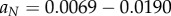
yr^−1^, i.e. 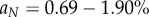.

**Table 1.** Estimations of the initial growth rate *a_N_* (in yr^−1^) of farmers in several regions, based on sum-calibrated probabilities of Funnel Beaker sites versus time (figs. 3 and 4 in [44]). For a map with these regions and their dated sites, see fig. 1 in [44]. That paper includes some additional regions to the south (in Germany and Poland), but we do not use them because they are outside the area implied by the database of sites that we have used to estimate the Neolithic spread rate in Scandinavia. If a plot displays several increases near the arrival of the Neolithic (6000 cal yr BP), we report the highest value of *a_N_* as an estimation of the maximum possible growth rate that could have driven the spread of the population front.

	whole set of dates^a^ (fig. 3 in [44])	only dates from settlements (fig. 4 in [44])
western Sweden	0.0069	0.0139
Skåne and Bornholm	0.0139	0.0110
northern Jutland	0.0087	0.0110
central southern Sweden	0.0118	0.0190
Swedish Baltic Isles	0.0125	0.0116
eastern middle Sweden	0.0105	0.0110
Danish Isles	0.0154	0.0146

^a^Settlements, enclosures, graves and ritual depositions ([44], p. 3332).

It is worth noting that the range for *a_N_* obtained in the previous paragraph is strictly an upper bound to demographic growth, because cultural transmission could have led to additional increases in the populations of farmers (besides those due to demographic growth). However, such additional effects are likely to be small. The reason for this is that if *C* hunter–gatherers are incorporated into the farming communities by each farmer per generation, then the equation 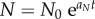 (used in equation (4.1)) becomes (for *t* = *T*) the more general equation 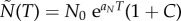 (see electronic supplementary material, equation (S18)) and the correction due to cultural transmission (in %) can be written as 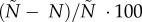, which is 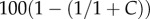 or below 2% for *C* = 0.02 [24] at *t* = 1 generation and 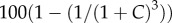 or below 6% at *t* = 3 generations (or about 100 yr). Therefore, this correction is likely to be small and this justifies using the range estimated above, i.e. 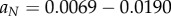 yr^−1^.

Besides the initial growth rate *a_N_*, to obtain a numerical value for the spread rate using equation (3.6) we need the dispersal kernel and the generation time. The following dispersal kernels have been estimated from ethnographic data of pre-industrial agriculturalists [37]:

Kernel A (Gilishi 15) in [37]: {*p_j_*}={0.54, 0.17, 0.04, 0.25}, {*r_j_*}={2.4, 14.5, 36.3, 60.4}km.
Kernel B (Gilishi 25) in [37]: {*p_j_*}={0.40, 0.17, 0.17, 0.26}, {*r_j_*}={2.4, 14.5, 36.3, 60.4}km.
Kernel C (Shiri 15) in [37]: {*p_j_*}={0.19, 0.07, 0.22, 0.52}, {*r_j_*}={2.4, 14.5, 36.2, 60.4}km.
Kernel D (Yanomamö) in [37]: {*p_j_*}={0.19, 0.54, 0.17, 0.04, 0.04, 0.02}, {*r_j_*}={5, 30, 50, 70, 90, 110}km.
Kernel E (Issongos) in [37]: {*p_j_*}={0.42, 0.23, 0.16, 0.08, 0.07, 0.02, 0.01, 0.01}, {*r_j_*}={2.3, 7.3, 15, 25, 35, 45, 55, 100}km.

These five dispersal kernels have been measured for pre-industrial farming populations. Such kernels are very difficult to find. For example, there are nineteenth to twentieth century kernels for Europe [47], but it is known that mechanized forms of transport led to a dramatic increase in dispersal in mid-nineteenth century Europe [48], which probably makes such kernels inappropriate to model the spread of the Neolithic.

For the generation time, we use the range 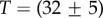 yr, as estimated with 95% CL from the frequency data for pre-industrial farmers reported in [45].

Unfortunately, we cannot use any range for the cultural diffusion intensity *C*, because it cannot be estimated with sufficient confidence from ethnographic observations (there are quantitative data for a few populations of farmers [5], but it is possible that in other populations no hunter–gatherers were incorporated (*C* = 0), and we do not know the percentage of populations with 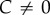 and *C* = 0). In fact, *C* can be estimated from ancient genetic data [24] but, as we shall explain (§5), such data are still too few in Scandinavia. Therefore, we will analyse the dependence of the Neolithic spread rate on the cultural diffusion intensity *C*, and find the range for *C* that is consistent with the observed spread rate.

## 5. Estimating the relative importance of demic and cultural diffusion

**Figure 3.**
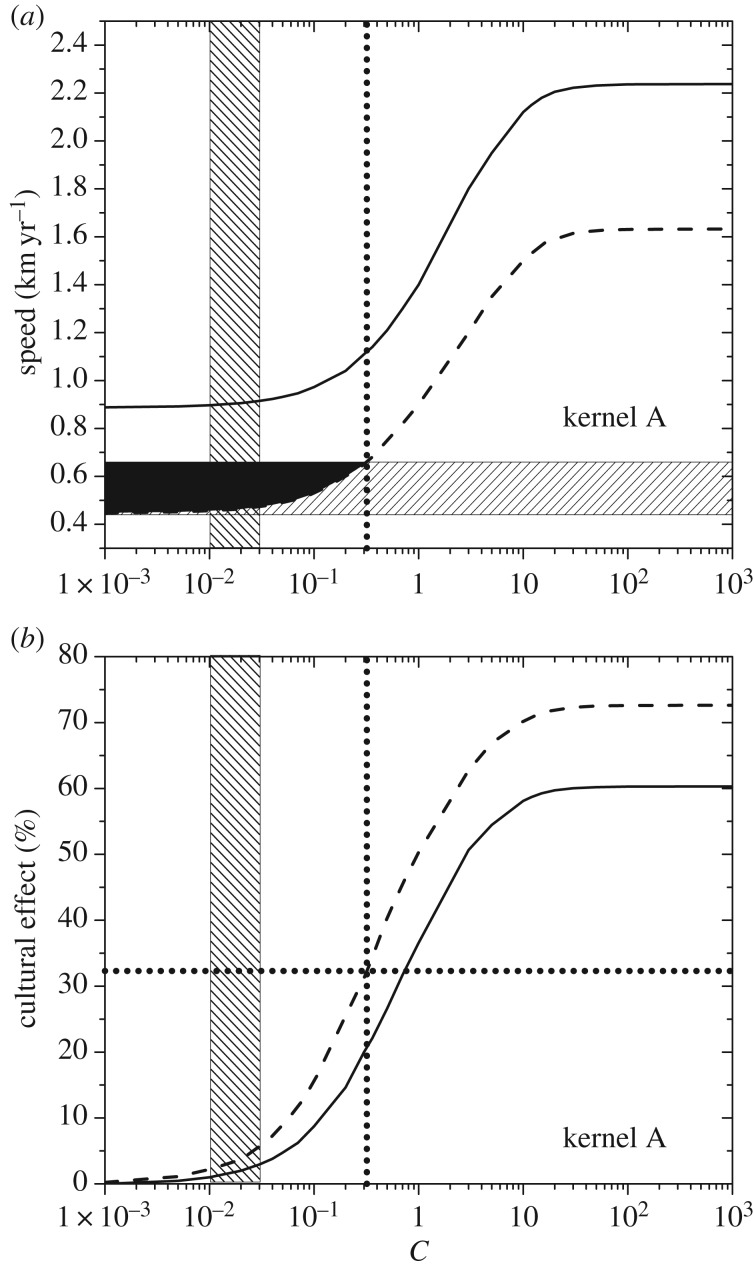
(*a*) The area between the two curves gives the spread rates predicted by the model for dispersal kernel A as a function of the cultural diffusion intensity *C*, and the horizontal hatched rectangle is the observed spread rate from the archaeological data. (*b*) The cultural effect (in per cent) as a function of the cultural diffusion intensity *C*, for the same curves as in (*a*). The horizontal dotted line gives the maximum cultural effect.

**Figure 4.**
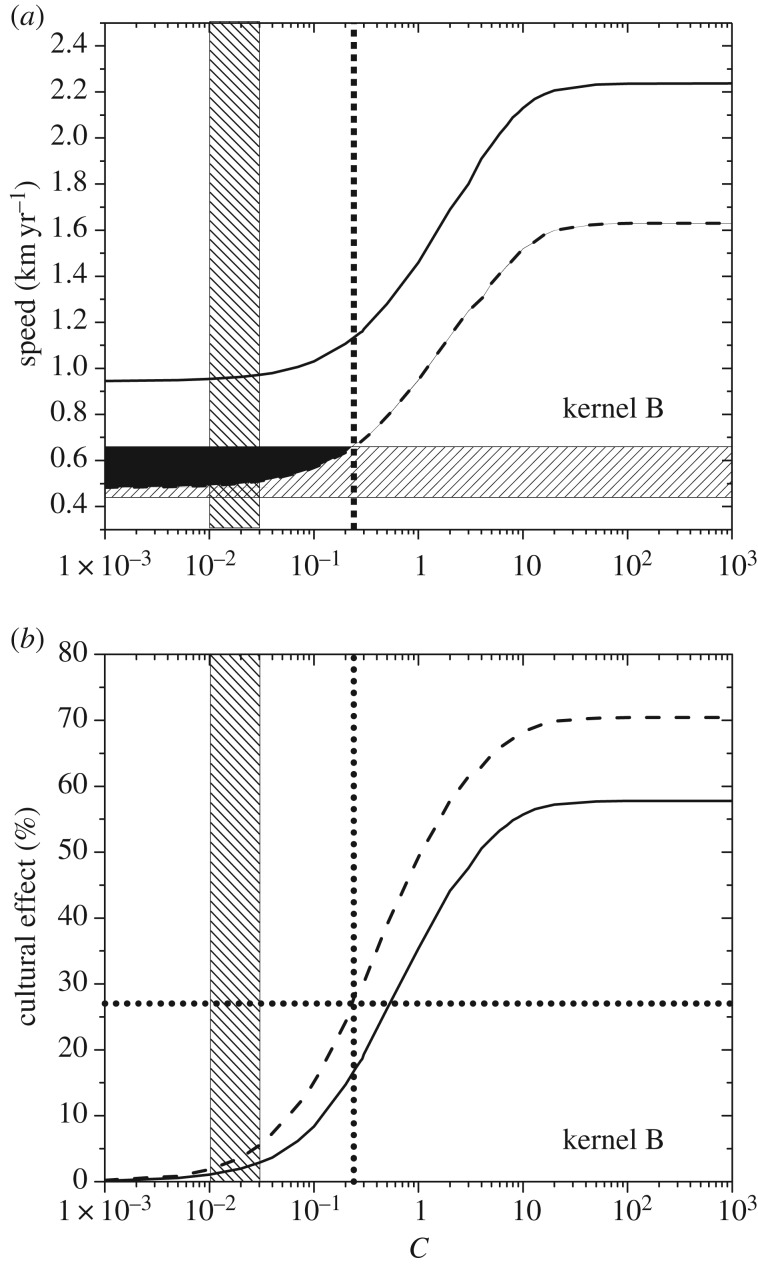
(*a*) The area between the two curves gives the spread rates predicted by the model for dispersal kernel B as a function of the cultural diffusion intensity *C*, and the horizontal hatched rectangle is the observed spread rate from the archaeological data. (*b*) The cultural effect (in per cent) as a function of the cultural diffusion intensity *C*, for the same curves as in (*a*). The horizontal dotted line gives the maximum cultural effect.

**Figure 5.**
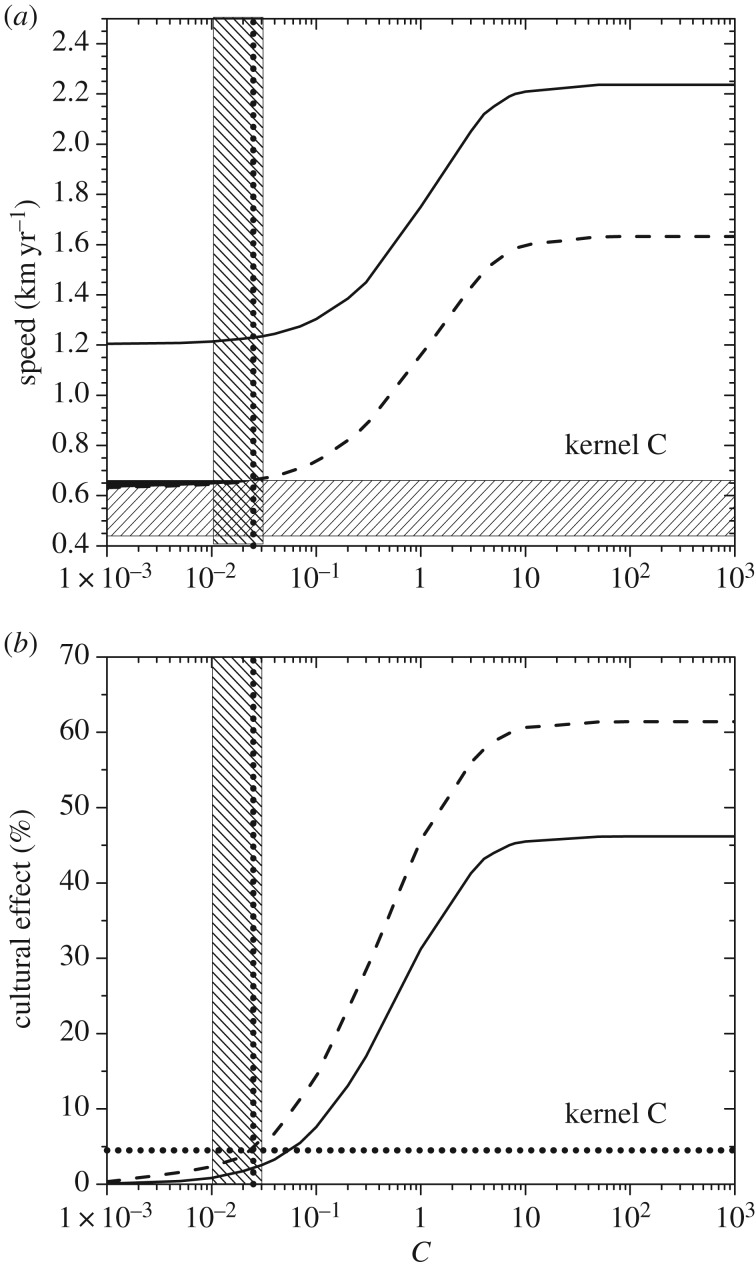
(*a*) The area between the two curves gives the spread rates predicted by the model for dispersal kernel C as a function of the cultural diffusion intensity *C*, and the horizontal hatched rectangle is the observed spread rate from the archaeological data. (*b*) The cultural effect (in per cent) as a function of the cultural diffusion intensity *C*, for the same curves as in (*a*). The horizontal dotted line gives the maximum cultural effect.

**Figure 6.**
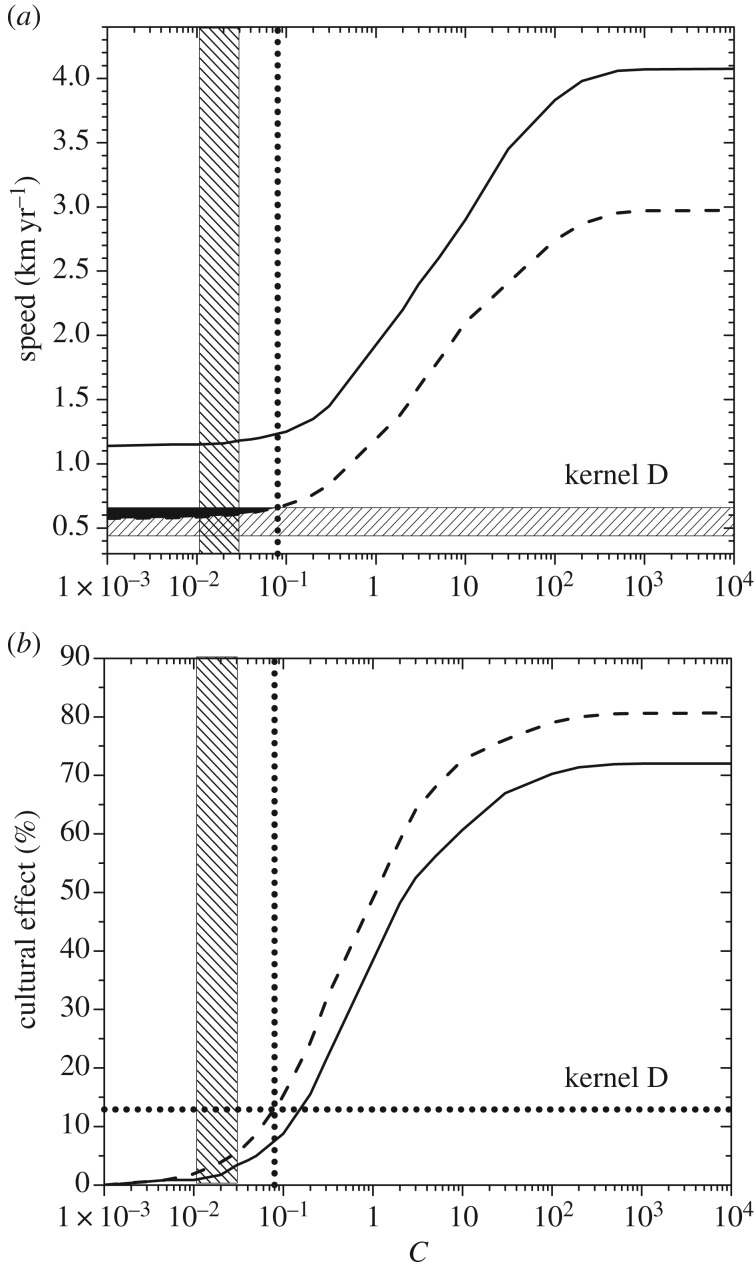
(*a*) The area between the two curves gives the spread rates predicted by the model for dispersal kernel D as a function of the cultural diffusion intensity *C*, and the horizontal hatched rectangle is the observed spread rate from the archaeological data. (*b*) The cultural effect (in per cent) as a function of the cultural diffusion intensity *C*, for the same curves as in (*a*). The horizontal dotted line gives the maximum cultural effect.

**Figure 7.**
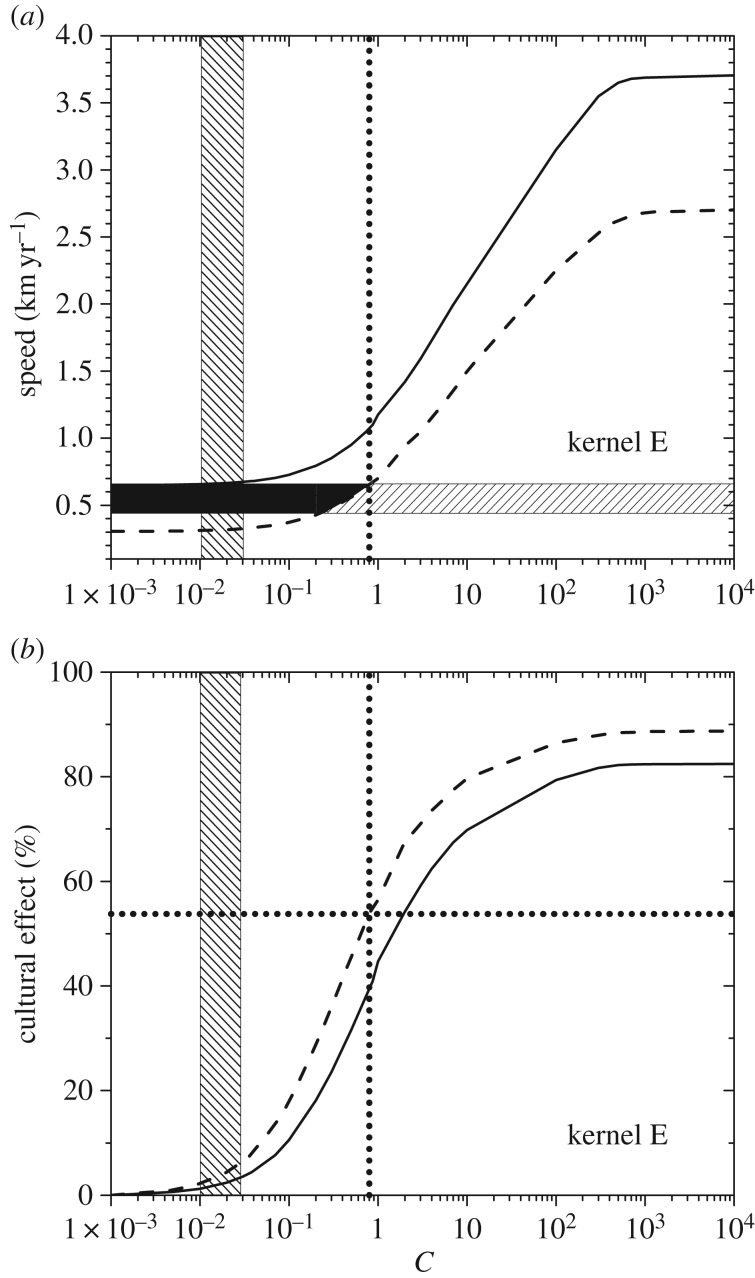
(*a*) The area between the two curves gives the spread rates predicted by the model for dispersal kernel E as a function of the cultural diffusion intensity *C*, and the horizontal hatched rectangle is the observed spread rate from the archaeological data. (*b*) The cultural effect (in per cent) as a function of the cultural diffusion intensity *C*, for the same curves as in (*a*). The horizontal dotted line gives the maximum cultural effect.

The two curves in figure 3*a* give, for dispersal kernel A, the maximum and minimum spread rates obtained from equation (3.6). The maximum spread rate (full curve) has been obtained by using the maximum value of the initial growth rate (*a* = 0.0190 yr^−1^) and the minimum value of the generation time (*T* = 27 yr; see §4). The minimum spread rate (dashed curve) has been obtained by using the minimum growth rate (*a* = 0.0069 yr^−1^) and the *maximum* generation time (*T* = 37 yr). The reason why the *maximum* generation time is used to obtain the *minimum* spread rate is the following. In §3, we have defined the generation time *T* as the mean time interval between the birth of a parent and the birth of one of her/his children. Equivalently, *T* is the mean time interval between the dispersal of a parent (usually when she/he mates) and the dispersal of one of her/his children (when the latter mates). Thus, *T* is the time interval between two successive dispersal events (obviously, additional movements after reproduction do not affect the population dynamics, so they are irrelevant concerning the spread of the wave of advance). Therefore, a larger value of *T* corresponds to a larger time interval between successive dispersal events, i.e. to a slower motion of the wave of advance. This is why the *maximum* generation time corresponds to the *minimum* spread rate, as mentioned above and applied in figure 3. Similar to figure 3, figures 4*a*–7*a* give the maximum and minimum spread rates using kernels B, C, D and E, respectively. The horizontal rectangle in figures 3*a–*7*a* corresponds to the observed range, i.e. 0.44–0.66 km yr^−1^ (which has been obtained from figure 2 in §2). Figures 3*b*–7*b* give the percentage of the cultural effect, which has been previously defined [5] as the difference between the spread rate (for the value of *C* considered) and the spread rate for *C* = 0 (purely demic diffusion), divided by the former and multiplied by 100. Thus, figure 3*b* has been obtained from the results shown in figure 3*a*; figure 4*b* has been obtained from figure 4*a*, etc.

In figures 3*a*–7*a*, the black area gives the pairs of values of the speed and *C* for which the speed from the archaeological data (horizontal hatched rectangle) is consistent with the speed predicted by the mathematical model (area between the two curves).

In figures 3*a*–7*a*, the vertical dotted line corresponds to the maximum value of *C* for which the model is consistent with the observed spread rate (black area). The same vertical dotted line appears in figures 3*b*–7*b*. In the latter figures, the horizontal dotted line gives the cultural effect (in per cent) for this value of *C*, i.e. the maximum possible value of the cultural effect.

In figures 3*a*–7*a*, we observe that for purely demic diffusion (*C* = 0) the observed speed (horizontal hatched rectangle) is always consistent with the mathematical model (i.e. the range between the two curves), independently of the dispersal kernel considered. However, considering purely demic diffusion (*C* = 0), we cannot obtain any maximum value for the cultural effect. We can solve this problem because we are using a demic–cultural model (§3) rather than a purely demic one [37]. Thus, we can estimate the cultural effect quantitatively using figures 3*b*–7*b*. In these figures, we observe that the maximum cultural effect on the spread rate depends strongly on the dispersal kernel. The smallest value is 5% (kernel C) and the largest one is almost 54% (kernel E), whereas the intermediate values are 13% (kernel D), 27% (kernel B) and 32% (kernel A). Interestingly, these percentages (maximum cultural effects) are always below 50% for kernels A, B, C and D. The only exception is kernel E (figure 7*b*), but even in this case the percentage is always below 54%. The cultural effect can thus be slightly above 50%, but only for kernel E and assuming extreme values of *C* and the speed, namely values of *C* very close to the upper bound implied the observed rate (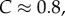 from figure 7*a*) and very slow speeds (close to the dashed line in figure 7*a,b*), i.e. assuming also very low reproduction rates *a_N_* and/or very high values of *T*. Thus, we can conclude that archaeological data clearly suggest that the cultural effect was below 50%, i.e. that demic diffusion had a more important effect than cultural diffusion on the Neolithic spread rate in Scandinavia.

Finally, we mention that it is unfortunate that at present there are so few ancient genetic data of early farmers in Scandinavia, so that we cannot compare any ancient genetic Scandinavian cline with our model. This would be useful to perform a more accurate estimation of the value of *C*, similar to what we have done recently for Europe [24]. The range for Europe is 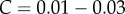 and is shown as a vertical hatched rectangle in figures 3–7. This indicates that, if a similar range for *C* were valid for Scandinavia, the Neolithic spread would have been mainly demic indeed. However, we stress that the value of *C* in Scandinavia can be different from the average value in Europe, and future analyses of ancient genetic clines for Scandinavia (similar to that in [24] for Europe) will hopefully be able to perform such an accurate estimation of the value of *C*, and hence of the cultural effect (when sufficient ancient genetic data for Scandinavia become available).

## 6. Conclusion

In this work, we have obtained a quantitative estimation of the spread rate of the Neolithic in Scandinavia. Some authors have argued qualitatively that it was rapid [27,28], but we are not aware of any previous quantitative estimation. We have found that the Neolithic spread rate in Scandinavia was 0.44–0.66 km yr^−1^, with 95% CL. This is substantially slower (by about 50%) than the spread rate across the Near East and Europe, namely 0.9–1.0 km yr^−1^ (95% CL), which was previously obtained by the same method (namely, a linear regression of calibrated dates versus great-circle distances) [34].

Why was the Neolithic spread rate in Scandinavia so slow compared with most of Europe? We have estimated values of the initial growth rate *a_N_* for populations of early farmers in Scandinavia, using plots of sum-calibrated probabilities of Funnel Beaker sites versus time (figs. 3 and 4 in [44]). In this way, we have obtained the range 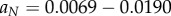
yr^−1^, i.e. 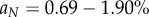 (table 1). Such values of *a_N_* are substantially lower than those previously estimated from ethnographic data and used to model the spread of the Neolithic in Europe (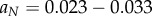
yr^−1^, i.e. 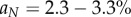) [5]. Using a new mathematical wave-of-advance model, we have seen quantitatively that the lower reproductive rates (*a_N_*) of farmers in Scandinavia explain the slower spread rate of the Neolithic (figures 3*a*–7*a*), as compared with most of Europe. Additional, indirect support for this possibility comes from ethnographic data, according to which modern human populations at higher latitudes tend to have lower reproduction rates [40]. Of course, modern reproduction rates cannot be directly applied to the Neolithic spread because present populations are close to saturation, whereas pioneering Neolithic populations had low population densities, and therefore faster reproduction rates (the logistic model, which is appropriate for many populations, including humans [49], displays fast population growth at low densities and diminishing net reproduction as the population density increases and approaches saturation [46]).

We have introduced a mathematical model (§3) that includes both horizontal and vertical cultural diffusion, besides demic diffusion. We have seen, by comparing the spread rate from the archaeological dates (horizontal rectangle in figures 3*a*–7*a*, obtained from figure 2) with the predictions of the model for five different dispersal kernels of pre-industrial famers (area between the two curves in figures 3*a*–7*a*), that the rate was dominated by demic diffusion, whereas cultural diffusion played a secondary role. This is clearly seen in figures 3*b*–7*b*, where the percentage of the cultural effect is always below 50% (the only exception is figure 7*b*, but even in this case the cultural effect is always below 54%, and is below 50% except for extreme parameter values).

From figures 3*a*–7*a*, we find that *C* < 0.8, which indicates that, on average, fewer than eight hunter–gatherers were incorporated in the Neolithic communities by each group of 10 pioneering farmers, via either vertical or horizontal cultural transmission.

Our new model (§3) can be applied to other instances of demic and cultural spread, not only of farming but also of other cultural traits.

Finally, we mention that some Early Neolithic parent–children pairs have been recently identified using genetic methods. However, for all pairs identified so far, the parent and the child are buried together [50]. If in the future geneticists could identify parent–children pairs such that the parent is buried in one place and the child in another place, it could be possible to estimate the dispersal kernel directly from archaeological (instead of ethnographic) data, and this would lead to more precise results. This improvement would be analogous to the fact that in this paper we have used archaeological (rather than ethnographic) data to estimate initial growth rates and used them, for the first time, in spread rate computations using a mathematical wave-of-advance model.

## Supplementary Material

Supplementary material

## Supplementary Material

Database

## Data accessibility

All data are available as an Excel file (database) in the electronic supplementary material.

## Dedication

This paper is dedicated to the memory of Luigi Luca Cavalli-Sforza (1922–2018), whose pioneering work on Neolithic wave-of-advance models [2,4] was the starting point of the research reported in our previous works [5,24,29,33,34–37,45,46] and in this paper.
