# Supplementary material for: Estimating the relative importance of demic and cultural diffusion in the spread of the Neolithic in Scandinavia
Source: J R Soc Interface. 2018 Nov 21;15(148):20180597. doi: 10.1098/rsif.2018.0597 (PMC6283996; doi:10.1098/rsif.2018.0597)
Supplement: Supplementary material [file rsif20180597supp1.pdf]

Fort , Pareta & Sørensen. Estimating the relative importance of demic and cultural diffusion in the spread of the Neolithic in Scandinavia. *J. R. S. Interface*

Supplementary material

Index

S1. The effect of landscape and vegetation 1

S2. Non-linear models 3

S3. Justification of the origin of distances 6

S4. Details on the estimation of the spread rate 8

S5. Details on the wave-of-advance model 8

S1. The effect of landscape and vegetation

In the main paper we have estimated distances using great circles. In other words, we have considered the minimum possible distance between any two points on the Earth surface. Certainly, this will not be an exact estimation in general, because the routes followed by migrating farmers may have been affected by factors such as landscape, vegetation, etc. We cannot assume an arbitrary cost function for such effects (e.g., we cannot simply duplicate the distance if the altitude or the vegetation duplicates), because this would use totally unjustified assumptions and/or parameter values. Moreover, without a careful justification, it would not be reasonable to assume that such cost-distances are measured in km. But we need distances measured in km for our purposes, namely to estimate the spread rate in km/yr. Thus we use an approach that we previously introduced and called 'shortest paths' in the study of the Neolithic spread in continental Europe [1]. Here we improve it by using the free internet application google maps (https://www.google.com/maps). Using this application, we can find the shortest-path route by foot between two geographical points (see Fig. S1 for an example). Obviously we cannot proof that Neolithic farmers followed similar paths to those used at present, but human communication routes by foot are very stable over time, and they have two additional advantages: (i) they certainly take into account landmass features (mountains, seas, etc.), as well as any other feature that might influence human travel (e.g., vegetation, soil type, roughness, etc.); and (ii) they yield distances measured in km.

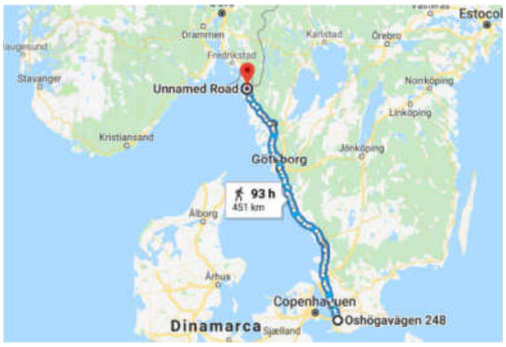

Fig. S1. Example of distance computation using google maps. The origin (lower circle) is the site of Oxie and the end (upper circle) is the site of Skee. For some other final locations (instead of Skee), google maps includes sea travel for part of the route, but the distances from the coast are small (<100 km) and therefore reasonable if we take into account the navigation capabilities of early farmers (as implied, e.g., by the fact that the Neolithic arrived to the island of Cyprus, which is about 100 km from the mainland). Although the mathematical model in the main paper does not strictly include sea travel (so that an equation for the spread rate can be found), it is reasonable to assume that it will be approximately valid for so small distances over sea. Another option would be to avoid sea travel in the computation of all distances, but this would contradict the fact that the Neolithic arrived to Sweden across the Baltic sea.

In Fig. S2 we show the regression using these shortest-path distances. Thus Fig. S2 is the same as Fig. 2 in the main paper, but using shortest paths instead of great circles. Obviously, the spread rate implied by the regression fit in Fig. S2 should be faster than that by Fig. 2 in the main paper, because there we have used the shortest possible distance between two points on the Earth surface (great circle). The spread rate implied by the regression fit in Fig. S2 is 0.56-0.84 km/yr, which is similar to that in the main paper (0.44-0.66 km/yr). Indeed, replacing the horizontal rectangle in Figs. 3-7 (main paper) by the spread range from Fig. S2 (0.56-0.84 km/yr), it is very easy to see that the implied percentage for the cultural effect is below 50% in all cases except Fig. 7. As explained in the main paper (Sec. 5), the same happens for the range found in the main paper (0.44-0.66 km/yr). Therefore, we conclude that using our procedure to take into account the effects of landscape, vegetation, etc., does not change the conclusions in the main paper.

Finally we would like to stress that clearly cost distances (here defined as road distances by foot obtained using google maps) are more realistic estimations of length than great circles for individual travels, but we should keep in mind the spread rate of a wave of advance is a macroscopic quantity (i.e., it is valid at scales sufficiently large compared to that of the individual displacements, i.e. to the distances in the dispersal kernel). In other words, we should distinguish between the effects of large and small obstacles. On one hand, large obstacles (compared to the distances in the dispersal kernel) can deflect the wave of advance and thus lead to higher distances (and a faster observed spread rate) than great circles. On the other hand, small obstacles (leading, e.g., to a few curves in a road) will affect the length of an individual travel, but we think that it is not realistic to expect that they will have any noticeable effect on the spread rate of the wave of advance or its direction (note that the vector (Δ_x, Δ_y) appearing in Eq. (3.1) or (S5) depends on the initial and final locations of each displacement, but not on

the detailed path). Therefore, in our opinion the validity of cost distances should not be overstated, and it is reasonable to consider that both great circles (main paper) and cost distances (this section) give reasonable bounds on the observed spread rate.

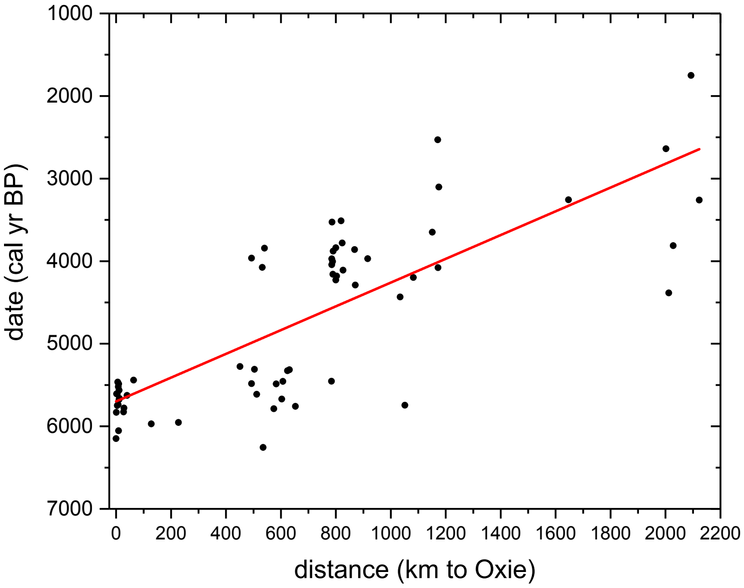

Fig. S2. Linear regression taking into account that distances are affected by landscape, vegetation, etc. According to this linear fit (full line), the spread rate of the Neolithic in Scandinavia was 0.56-0.84 km/yr (r = 0.78), which is quite similar to the results in the main paper (Fig. 2) using great-circle distances (namely, 0.44-0.66 km/yr and r = 0.77). In both cases N = 63 sites and the origin of distances is Oxie, the oldest site in the database.

S2. Non-linear models

In the main paper (Fig. 2), we have fitted a linear dependency ($y = a + bx$) to the oldest dates of Neolithic sites (variable y) versus their distances from a putative origin of spread (variable x). In this section, we consider more complicated (i.e., non-linear) dependencies, and justify that they are not necessary for our purposes.

The Pearson correlation coefficient, r, is defined so that it square is given by [2]

$$r^2 = 1 - \frac{\sum_{i=1}^{N}(y_{est\ i} - y_i)^2}{\sum_{i=1}^{N}(y_i - \bar{y})^2}, \quad \text{(S1)}$$

where the index $i = 1,2,3,\ldots,N$ identifies the site, y_i is its observed date (defined as the oldest of all dates obtained by radiocarbon dating), $\bar{y}$ is the average date over all sites ($\bar{y} = \sum_{i=1}^{N} y_i/N$), and $y_{est\ i}$ is

the date estimated (by the model considered) for site i. If the model produced for all sites exactly their observed dates ($y_{est\ i} = y_i$ for all i), the numerator in Eq. (S1) would vanish and $r = 1$. Otherwise, the better the agreement between the model and the data, the lower the value of the numerator in Eq. (S1) and the higher the value of r. According to linear regression theory [2], $0 \leq r \leq 1$.

Let us first consider, instead of a linear model ($y_{est} = a + bx$), a second-order polynomial ($y_{est} = a + bx + cx^2$). This implies to include an additional parameter (c) that will be fitted to obtain the best possible agreement between the model and the data. Thus the values $y_{est\ i}$ obtained from the second-order model will tend to be closer to the data y_i than for the linear model, i.e., the values of $y_{est\ i} - y_i$ will tend to be smaller in magnitude, and Eq. (S1) implies that the value of r will be higher than for the linear model (except if $c = 0$, but in this case the value of r will remain the same because the polynomial becomes of first order, i.e. we would be dealing again with the linear model). Thus, considering a model with more parameters will never decrease the value of r. Therefore, we cannot use the Pearson correlation coefficient r to determine which model is better. Instead, according to statistical theory, the so-called adjusted correlation coefficient should be used. Its square is given by [2]

$$r_{adj}^2 = 1 - \frac{(1-r^2)(N-1)}{(N-p)}, \qquad \text{(S2)}$$

where p is the number of parameters in the model considered. Note that r_{adj} is the same as the Pearson correlation coefficient r if $p = 1$, i.e. if the model has only one parameter (this happens, e.g., for the model $y_{est} = bx$). According to Eq. (S2), increasing the value of p tends to decrease the value of r_{adj}, but obviously this also leads to a new fitted function y_{est}, so according to Eq. (S1) the value of r^2 will also change. In general, it is justified to increase the value of p (number of parameters in the model) if r^2 diminishes sufficiently to cause an increase in the value of r_{adj}, given by Eq. (S2) [2].

The results for the data considered in the main paper are as follows. For the linear model ($y_{est} = a + bx$, Fig. 2 in the main paper), $r_{adj} = 0.7705$. For the second-order model ($y_{est} = a + bx + cx^2$), $r_{adj} = 0.7739$. The increase in r_{adj} is very small (below 1%). This suggests that there is no point in complicating the model by including the second-order term (cx^2). This can be confirmed by visual inspection of the two models or, more rigorously, by computation of the spread rates implied by them, as we explain in the next paragraph.

In Fig. S3 we show the regression fits according to the linear and second-order models. It is seen that both models yield essentially the same dependency. The deviation at distances above 1,200 km is likely due to the fact that there are only 5 dated sites in this range. Indeed, in the range 0-1,200 km the spread rate (inverse of the slope) implied by the second-order model (blue line in Fig. S3) is 0.41-0.70 km/yr. This is very similar to the range estimated from the linear model in the main paper, namely 0.44-0.66 km/yr with 95% confidence level (CL) or 0.40-0.70 km/yr with 99% CL (black line in Fig. S3 or Fig. 2 in the main paper). Therefore, we can be sure that all of the conclusions of the main paper would be the same if we used the second-order model instead of the linear one.

Because there is no substantial increase in r_{adj}, and also because the results would not change, there is no point in complicating the model by including a second-order term. This is why we have used the linear model in the main paper. Below we discuss two other non-linear models and arrive at the same conclusion.

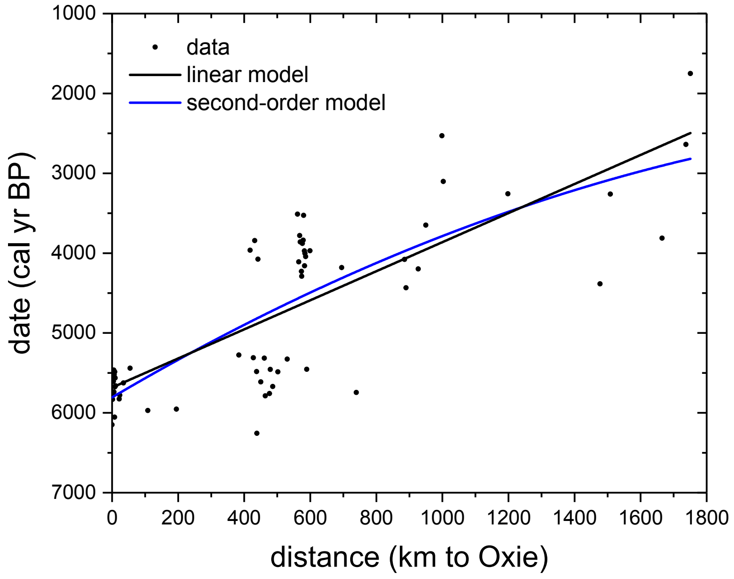

Fig. S3. Linear (i.e. first-order) and second-order fits to the data. The linear fit is the same as in Fig. 2 in the main paper.

We have also considered a third-order polynomial model ($y_{est} = a + bx + cx^2 + dx^3$). Again, the value of r_{adj} is almost the same as for the first and second-order models above. In fact $r_{adj} = 0.7702$, which implies that r_{adj} does not increase but decreases, so there is surely no improvement in this case (not even a negligible one).

Finally we consider an exponential model ($y_{est} = ae^{bx}$). We see in Fig. S4 that the behavior is again almost linear. In this case, $r_{adj} = 0.7778$, so the increase relative to the linear model ($r_{adj} = 0.7705$) is again below 1%. Moreover, we can estimate the spread rate by computing the inverse of the slope of the exponential model (red line) in Fig. S4 in the distance range 0-1200 km/yr (as done above for the second-order model). The result is 0.41-0.68 km/yr, once more very similar to the range estimated from the linear model in the main paper (0.44-0.66 km/yr with 95% CL, or 0.40-0.70 km/yr with 99% CL). Therefore, we can be confident that all of the conclusions of the main paper would not change if we considered an exponential model rather than a linear one.

For all three non-linear models tested, complicating the linear model leads to negligible changes in the value of r_{adj}. Clearly, this indicates that there is no point in considering non-linear models. As

explained above, this is also confirmed by the corresponding ranges of the spread rate, which are almost the same as for the linear model and would, therefore, lead to the same conclusions as those reported in the main paper.

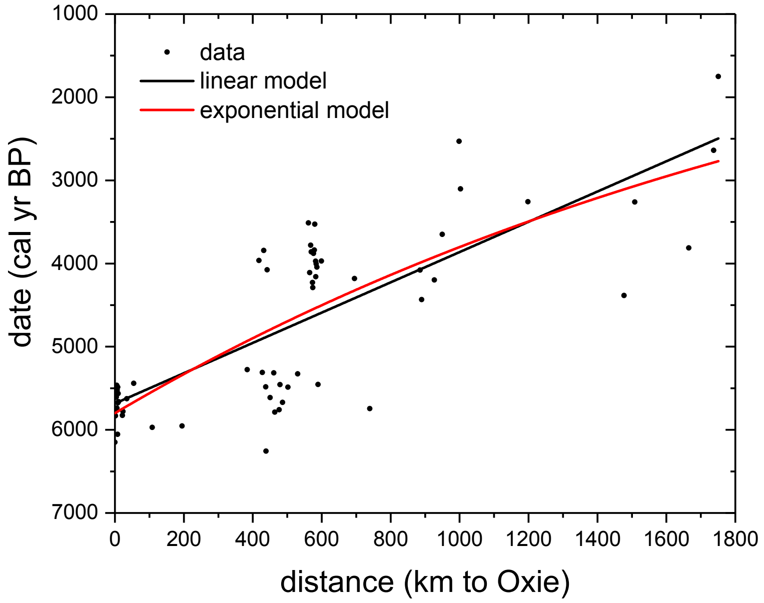

Fig. S4. Linear and exponential fits to the data. The linear fit is the same as in Fig. 2 in the main paper.

S3. Justification of the origin of distances

In the main paper, we have used the site of Oxie (4,200 cal yr BC) as the origin of distances, because it is the oldest site in the database. Here we check statistically that Oxie is a reasonable origin, by comparing the corresponding value of the Pearson correlation coefficient r to those using other origins. Alternatively, instead of the Pearson correlation coefficient r, other measures could be used (e.g., Akaike's Information Criterion, AIC). However, simulated dispersals have shown than those indices are maximized for the same origin as r provided that $r > 0.3$. This is indeed our case, because in the main paper we have found $r > 0.7$ assuming Oxie as origin. Therefore, for our purposes it is justified to use the Pearson correlation coefficient r.

We defined a square grid with nodes separated 1° latitude and 1° longitude, covering the whole area in Fig. 1 in the main paper. We considered all nodes not located on the sea as possible origins of the Neolithic spread. For each origin, we computed the distance from it to all 70 sites in our database, and used these distances to find the linear regression of the dates of all sites versus these distances. In this

way, we obtained the Pearson correlation coefficient r for each origin or node. Figure S5 shows an interpolation of these values of r. Similarly to previous work [3, 4], we identify the most likely area for the location of the origin of the dispersal. This is the red area in Fig. S5, which has the highest values of r for our database. As mentioned in the main paper, the site of Oxie is located near the coast of the southern tip of Sweden, so it falls within the red area in Fig. S5. Therefore, it is clearly reasonable to use Oxie as origin of distances. Alternatively, we could use as origin the only node within the red area in Fig. S5 that has a value of r higher than Oxie, namely the point with coordinates (56N, 14E). Using all 70 sites, this would yield $r = 0.799$. The difference in the value of r relative to Oxie ($r = 0.792$) is very small (below 1%). Moreover, the spread range using the point (56N, 14E) as origin is 0.39-0.57 km/yr. This is very similar to that estimated using Oxie as origin in the main paper (0.42-0.62 km/yr, also with 70 sites), so we can be sure that the conclusions of our paper would not change (in fact they would be strengthened, because a slower observed speed corresponds to a lower cultural effect).

For completeness we mention that, if we preferred to use only 63 sites (instead of all 70 sites in the database), i.e., if we excluded the 7 sites in Denmark and Finland (as explained in the main paper), the results would be 0.40-0.60 km/yr ($r = 0.786$) for the origin at (56N, 14E), versus 0.44-0.66 km/yr for the origin at Oxie ($r = 0.775$) Again both ranges are so similar that the conclusions of our paper would be the same (in fact they would be strengthened if using the origin at (56N, 14E), for the reason given in the last sentence of the previous paragraph).

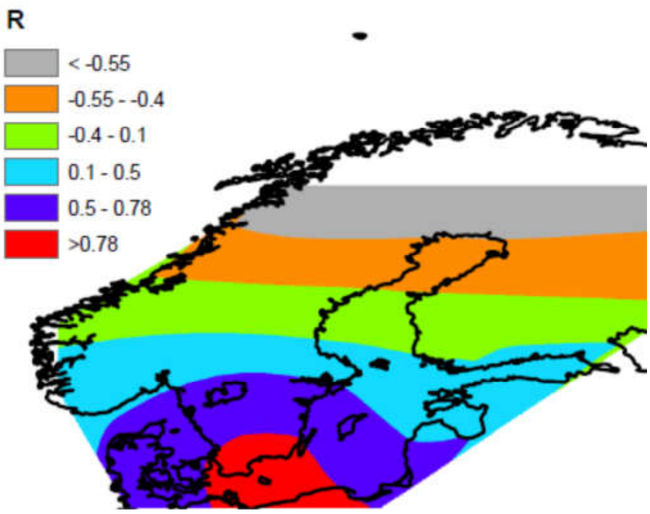

Fig. S5. Interpolation of the values of the correlation coefficient using 70 sites and the nodes of a grid as possible origins.

S4. Details on the estimation of the spread rate

The great-circle distance between two locations i and j as a function of their geographical coordinates (latitude φ and longitude λ) is given by the Haversine equation [5],

$$d_{ij} = 2R \arcsin\left(\sqrt{\sin^2\left(\frac{\varphi_i - \varphi_j}{2}\right) + \sin^2\left(\frac{\lambda_i - \lambda_j}{2}\right)\cos(\varphi_i)\cos(\varphi_j)}\right) \quad \text{(S1)}$$

where R is the mean Earth radius (R =6371 km).

As in previous work [6], we consider time as the dependent variable because it has presumably more error than distance (see main paper, Sec. 2). Therefore, the spread rate (in km/yr) is obtained as the inverse of the slope of the time-space regression, i.e.

$$speed = \frac{1}{slope}, \quad \text{(S2)}$$

and the standard error of this spread rate is, applying error propagation theory [7],

$$\sigma_{speed} = \frac{\sigma_{slope}}{(slope)^2}, \quad \text{(S3)}$$

where σ_{slope} is standard error of the slope. We have used Eqs. (S2) and (S3) to compute the 95% confidence-level (CL) interval for the spread rate, i.e., the range [8, 9]

$$(speed - t\sigma_{speed}, speed + t\sigma_{speed}), \quad \text{(S4)}$$

where t is Student's t-distribution for a 95% CL and $N - 2$ degrees of freedom, and N is the number of sites. In the present work $N \geq 63$ and therefore $t \approx 2$ [8, 9].

S5. Details on the wave-of-advance model

The evolution of the population densities of farmers N and hunter-gatherers P is driven by Eqs. (3.1)-(3.5) in the main paper. Here we explain these equations in more detail and derive the speed of the wave of advance. Eqs. (3.1) in the main paper are

$$\begin{cases} N(x,y,t+T) = \int_{-\infty}^{\infty}\int_{-\infty}^{\infty} \tilde{N}(x+\Delta_x, y+\Delta_y, t)\, \phi_N(\Delta_x, \Delta_y)\, d\Delta_x\, d\Delta_y \\ P(x,y,t+T) = \int_{-\infty}^{\infty}\int_{-\infty}^{\infty} \tilde{P}(x+\Delta_x, y+\Delta_y, t)\, \phi_P(\Delta_x, \Delta_y)\, d\Delta_x\, d\Delta_y, \end{cases} \quad \text{(S5)}$$

where $\phi_l(\Delta_x, \Delta_y)$ is the dispersal kernel of population l =N, P. The integrals in Eqs. (S5) cover the whole space because, in general, humans can move arbitrary distances and directions. However, below Eq. (S14) and in the main paper we consider specific dispersal kernels, so that in practice the distances and probabilities of motion are not arbitrary.

In Eqs. (S5) we have introduced

$$\begin{cases} \tilde{N}(x,y,t) \equiv R_T[N(x,y,t)] + H_T[N(x,y,t),P(x,y,t)] + V_T[N(x,y,t),P(x,y,t)] \\ \tilde{P}(x,y,t) \equiv R_T[P(x,y,t)] - H_T[N(x,y,t),P(x,y,t)] - V_T[N(x,y,t),P(x,y,t)], \end{cases} \tag{S6}$$

which account for the effects of reproduction (first term) and cultural transmission (last two terms).

The reproductive terms in Eqs. (S6) are given by logistic functions with initial growth rates a_l and carrying capacities K_l, namely

$$\begin{cases} R_T[N(x,y,t)] = \frac{e^{a_N T} K_N N(x,y,t)}{K_N + (e^{a_N T} - 1) N(x,y,t)} \\ R_T[P(x,y,t)] = \frac{e^{a_P T} K_P P(x,y,t)}{K_P + (e^{a_P T} - 1) P(x,y,t)}. \end{cases} \tag{S7}$$

For low population densities, Eqs. (S7) reduce to exponential growth (e.g., without dispersal $N(x,y,t+T) = R_T[N(x,y,t)] \approx e^{a_N T} N(x,y,t)$). As the population grows, net reproduction slows down until the carrying capacity is reached (e.g., $N(x,y,t) = K_N$). We use the logistic Eqs. (S7) because they agree with many observed data [10].

Horizontal transmission (acculturation) is given by

$$H_T[N(x,y,t),P(x,y,t)] = f \frac{R_T[N(x,y,t)]\, R_T[P(x,y,t)]}{R_T[N(x,y,t)] + \gamma\, R_T[P(x,y,t)]}, \tag{S8}$$

where the product $R_T[N(x,y,t)]\, R_T[P(x,y,t)]$ follows from the following reasoning [11, 12]. After reproduction, there are $R_T[N(x,y,t)]$ farmers and $R_T[P(x,y,t)]$ hunter-gatherers. If a hunter-gatherer (HG) contacts n teachers during his lifetime, then the number of his/her teachers who are farmers is nu, with $u = \frac{R_T[N(x,y,t)]}{R_T[N(x,y,t)] + R_T[P(x,y,t)]}$. If g is the probability that the HG becomes a farmer due to contact with a single farmer-teacher, the probability that the HG will become a farmer after n contacts is $1 - (1-g)^{nu}$. If g is small, this simplifies to fu, with $f = ng$. Thus, the total number of HGs becoming farmers per generation is $fu\, R_T[P(x,y,t)] = f \frac{R_T[N(x,y,t)]\, R_T[P(x,y,t)]}{R_T[N(x,y,t)] + R_T[P(x,y,t)]}$. The additional parameter γ in Eq. (S8) takes into account that a HG can have different probabilities to learn from other HGs than from farmers [12].

Finally, the term accounting for vertical transmission (interbreeding) in Eq. (S6) is given by

$$V_T[N(x,y,t),P(x,y,t)] = \eta \frac{R_T[N(x,y,t)] R_T[P(x,y,t)]}{R_T[N(x,y,t)] + R_T[P(x,y,t)]}. \tag{S9}$$

This equation follows from the fact that cultural transmission theory [11, 13] has shown by that the probability that a farmer mates a HG is proportional to the frequency of HGs, i.e. $\eta \frac{R_T[P(x,y,t)]}{R_T[N(x,y,t)] + R_T[P(x,y,t)]}$. Under random mating $\eta = 1$, but members of two populations with substantially different cultures (farmers and HGs in our case) obviously do not mate at random. Multiplying this probability by the number of farmers, i.e., $R_T[N(x,y,t)]$, yields the number of HGs that become farmers due to interbreeding, i.e., Eq. (S9) [13].

We apply the usual mathematical approach (linearization method) [14] to derive the front speed by considering a region where the pioneering populations of farmers arrive but hunter-gatherers are still close to their carrying capacity, i.e.

$$\begin{cases} N(x,y,t) = \varepsilon(x,y,t) + O(2) \\ P(x,y,t) = K_P - \delta(x,y,t) + O(2) \end{cases} \tag{S10}$$

where $\varepsilon(x,y,t) \ll K_N$, $\delta(x,y,t) \ll K_P$, and $O(2)$ are second-order terms. Then Eqs. (S7) simplify to

$$\begin{cases} R_T[N(x,y,t)] = e^{a_N T}\varepsilon(x,y,t) + O(2) \\ R_T[P(x,y,t)] = K_P - \frac{\delta(x,y,t)}{e^{a_P T}} + O(2), \end{cases} \tag{S11}$$

the first of Eqs. (S6) becomes

$$\widetilde{N}(x,y,t) = e^{a_N T}\left(1 + \frac{f}{\gamma} + \eta\right) N(x,y,t) + O(2) \tag{S12}$$

and the first of Eqs. (S5) simplifies to

$$N(x,y,t+T) \approx e^{a_N T}\left(1 + \frac{f}{\gamma} + \eta\right) \int_{-\infty}^{\infty}\int_{-\infty}^{\infty} N(x+\Delta_x, y+\Delta_y, t)\, \phi_N\left(\Delta_x, \Delta_y\right) d\Delta_x\, d\Delta_y. \tag{S13}$$

From Fig. 1 in the main paper, it is obvious that data are not detailed enough to measure different rates in different regions or directions. Therefore, there is no need to complicate the model with such features, and we can simply assume that all parameter values (and thus the speed) are uniform and isotropic. Then the easiest way to compute the speed is to do so along the x-axis ($y = 0$), by considering a coordinate frame $z = x - st$ moving with the wave of advance (we have introduced the symbol s for the spread rate or front speed). In order to find the speed we have to assume, as usual [14], that the population density decreases exponentially at large distances, $N(x,y,t) \propto \exp[-\lambda z] \to 0$ for $z \to \infty$ (with $\lambda > 0$). In this way, Eq. (S13) yields

$$e^{\lambda s T} = e^{a_N T}\left(1 + \frac{f}{\gamma} + \eta\right) \int_0^{2\pi} d\theta \int_0^{\infty} e^{-\lambda\Delta cos\theta}\, \phi_N(\Delta)\, \Delta\, d\Delta, \tag{S14}$$

where we have applied polar coordinates ($\Delta = \sqrt{\Delta_x^2 + \Delta_y^2}$, $\theta = tan^{-1}\frac{\Delta_y}{\Delta_x}$, and $d\Delta_x\, d\Delta_y = \Delta\, d\Delta\, d\theta$), so that the angle θ is defined so that $\Delta_x = \Delta \cos\theta$.

When dispersal data are measured, they are grouped in histograms or kernels *per unit length,* $\varphi_N(\Delta)$, defined as the probability to disperse into a ring of radius Δ and width $d\Delta$, divided by $d\Delta$. Therefore, if the data indicate that N-individuals (i.e., farmers) have probabilities p_j to disperse at distances r_j *(j=1,2,...,M),* we can write that

$$\varphi_N(\Delta) = \sum_{j=1}^{M} p_j\, \delta^{(1)}(r_j),$$

where $\delta^{(1)}(r_j)$ is the 1D Dirac delta centered at r_j (i.e., a function that vanishes everywhere except at $\Delta = r_j$). Obviously the total probability must be one,

$$1 = \int_0^{\infty} \varphi_N\,(\Delta)\, d\Delta,$$

and $\varphi_N(\Delta)$ is clearly a probability *per unit length.* In contrast, the kernel $\phi_N(\Delta_x, \Delta_y)$ used up to now is a probability *per unit area* (because in Eq. (S5) it is multiplied by $d\Delta_x\, d\Delta_y$, which has units of area). Therefore,

$$1 = \int_{-\infty}^{\infty}\int_{-\infty}^{\infty} \phi_N\left(\Delta_x, \Delta_y\right)\ d\Delta_x\, d\Delta_y = 2\pi \int_0^{\infty} \phi_N(\Delta)\ \Delta\ d\Delta,$$

where we have assumed the kernel is isotropic, $\phi_N(\Delta_x, \Delta_y) = \phi_N(\Delta)$ (because there are no ethnographic data detailed enough to detect anisotropies). Comparing the former two equations, we see that the dispersal probability per unit length $\varphi_N(\Delta)$ is related to that per unit area $\phi_N(\Delta)$ as [14]

$$\varphi_N(\Delta) = 2\pi\Delta\ \phi_N(\Delta)$$

and thus

$$\phi_N(\Delta) = \sum_{j=1}^{M} p_j \frac{\delta^{(1)}(r_j)}{2\pi\Delta}\ .$$

Finally, according to linear stability analysis, the minimum speed is that of the front [15]. Therefore, we obtain from Eq. (S14) that the spread rate of the farming waves of advance driven by Eqs. (S5)-(S9) is

$$s = \min_{\lambda>0} \frac{a_N T + \ln\left[(1+C)\left(\sum_{j=1}^{M} p_j I_0(\lambda r_j)\right)\right]}{T\lambda}, \tag{S15}$$

where

$$C = \frac{f}{\gamma} + \eta \tag{S16}$$

can be considered as a measure of the joint intensity of horizontal and vertical cultural transmission because, without net reproduction ($a_N = 0$) neither dispersal (i.e., replacing the integrals by $N(t)$), Eq. (S13) becomes simply

$$N(t+T) \approx (1+C)N(t), \tag{S17}$$

so C is equal to the mean number of hunter-gatherers converted into farming (by horizontal and/or vertical transmission) per pioneering farmer and generation.

$I_0(\lambda r_j) = \frac{1}{2\pi}\int_0^{2\pi} d\theta \exp[-\lambda r_j \cos\theta]$ in Eq. (S15) is the modified Bessel function of the first kind and order zero.

As mentioned above, dispersal data are usually reported using histograms, in which the quantity p_j (which appears in Eq. (S15)) is the probability for farmers to disperse a distance r_j, which is the mean distance of the corresponding histogram bin, and each bin is identified by the index $j = 1,2, \ldots, M$.

This completes the derivation of Eqs. (3.6) and (3.7) in the main paper (i.e., Eqs. (S15) and (S16)).

Without vertical transmission ($\eta = 0$), Eqs. (S15)-(S16) reduce to the result previously derived for horizontal transmission (Eq. [5] in Ref. [12]), as it should. Similarly, without horizontal transmission ($f = 0$), Eqs. (S15)-(S16) reduce to the result previously derived for vertical transmission and a special dispersal kernel (Eq. (54) in Ref. [13]), as it should.

Without dispersal (i.e., replacing the integrals by $N(t)$), Eq. (S13) becomes simply

$$N(t+T) \approx e^{a_N T}(1+C)N(t), \tag{S18}$$

which has been applied in the main paper, Sec. 4.

Finally we note that the assumption that space is homogeneous, in the sense that humans can live everywhere on the landscape, is necessary to obtain Eq. (S15), because otherwise it would not be possible to perform the integrals in Eqs. (S14) analytically.

References

[1] Pinhasi R., Fort J., Ammerman A. J., "Tracing the origin and spread of agriculture in Europe," *PLoS Biology,* vol. 3, no. e410, pp. 2220-2228, 2005.

[2] Draper, N. R., Smith, H., Applied regression analysis, New York: John Wiley and sons, 1981.

[3] Manica A., Amos W., Balloux F., Hanihara T., "The effect of ancient population bottlenecks on human phenotypic variation," *Nature,* vol. 448, pp. 346-349, 2007.

[4] Atkinson Q. D. , "Phonemic diversity supports a serial founder effect model of language expansion from Africa," *Science,* vol. 332, pp. 346-349, 2011.

[5] R. W. Sinnott, "Virtues of the Haversine," *Sky and Telescope,* vol. 68, p. 159, 1984.

[6] J. Fort, T. Pujol and L. L. Cavalli-Sforza, "Palaeolithic populations and waves of advance," *Cambridge Archaeol. J.,* vol. 14, pp. 53-61, 2004.

[7] J. Taylor, Introduction to error analysis, the study of uncertainties in physical measurements., Sausalito: University Science Books, 1997.

[8] M. R. Spiegel, J. J. Schiller, R. A. Srinivasan and M. LeVan, Probability and statistics, New York: McGraw-Hill, 1975.

[9] N. R. Draper and H. Smith, Applied Regression Analysis, New York: Wiley, 1981.

[10] A. J. Lotka, Elements of mathematical biology, New York: Dover, 1956.

[11] L. Cavalli-Sforza and M. Feldman, Cultural transmission and evolution: a quantitative approach, Princeton: Princeton University Press, 1981.

[12] J. Fort, "Synthesis between demic and cultural diffusion in the Neolithic transition in Europe," *Proc. Natl.*

Acad. Sci. U.S.A., vol. 109, pp. 18669-18673, 2012.

[13] J. Fort, "Vertical cultural transmission effects on demic front propagation: Theory and application to the Neolithic transition in Europe," *Phys. Rev. E,* vol. 83, no. 056124, 2011.

[14] J. Fort and P. Pujol, "Progress in front propagation research," *Rep. Progr. Phys.,* vol. 71, no. 086001, 2008.

[15] W. van Saarloos, "Front propagation into unstable states," *Phys. Rep.,* vol. 386, pp. 29-222, 2003.
